# Current pharmacotherapy of overactive bladder

**DOI:** 10.1590/S1677-5538.IBJU.2021.99.12

**Published:** 2021-05-10

**Authors:** Evgenyi I. Kreydin, Cristiano M. Gomes, Francisco Cruz

**Affiliations:** 1 University of Southern California Keck School of Medicine Department of Urology Los AngelesCA USA Department of Urology, Keck School of Medicine of the University of Southern California, Los Angeles, CA, USA; 2 Faculdade de Medicina da Universidade de São Paulo Departamento de Cirurgia Divisão de Urologia São PauloSP Brasil Divisão de Urologia, Departamento de Cirurgia, Faculdade de Medicina da Universidade de São Paulo, São Paulo, SP, Brasil; 3 Faculdade de Medicina do Porto Hospital de S. João Departamento de Urologia Porto Portugal Departamento de Urologia, Hospital de S. João, Faculdade de Medicina do Porto, Porto, Portugal; 4 i3S Instituto para Investigação e Inovação em Saúde Porto Portugal i3S Instituto para Investigação e Inovação em Saúde, Porto, Portugal

**Keywords:** Urinary Bladder, Overactive, Drug Therapy, Muscarinic Antagonists, Urinary Incontinence

## Abstract

Overactive bladder is a symptom complex consisting of bothersome storage urinary symptoms that is highly prevalent among both sexes and has a significant impact on quality of life. Various antimuscarinic agents and the beta-3 agonists mirabegron and vibegron are currently available for the treatment of OAB. Each drug has specific pharmacologic properties, dosing schedule and tolerability profile, making it essential to individualize the medical treatment for the patient's characteristics and expectations. In this manuscript, we review the most important factors involved in the contemporary pharmacological treatment of OAB.

## BACKGROUND

Overactive bladder is a symptom complex consisting of bothersome storage urinary symptoms, such as urinary frequency, urgency and nocturia in the absence of other (e.g. neurological) conditions (1). These symptoms may be associated with urgency urinary incontinence, resulting in the designation of “wet” OAB. Although there is no limit on the number of voiding or incontinence episodes, OAB is generally characterized by frequent, small-volume voids accompanied by urinary urgency.

OAB is a highly prevalent condition among both sexes, although most evidence suggests that a higher proportion of women than men suffer from this condition (2, 3). This difference is particularly pronounced for “wet” OAB, i.e., when urge urinary incontinence is present (4). The risk of developing OAB clearly increases with age but the overall prevalence seems to hover around 20% of the general population. Some geographic variability is present, but this effect is likely due to variation in study definitions and methodologies (5–7).

Studies have shown that OAB has a negative impact on the daily activities of affected individuals, with the potential to impair multiple domains of quality of life (QoL), including restriction of social and work life, while also resulting in higher healthcare resource use and costs (8–11). Despite the impact of OAB on QoL, treatment-seeking behavior is considered low, with rates varying from 14.6% to 43.6% (11–13).

When a patient presents with symptoms of OAB, it is important for the provider to identity underlying conditions that can lead to these symptoms so that they can be primarily addressed. A detailed history focusing on such factors as, benign prostatic hyperplasia in men, neurological disease, previous abdominal and pelvic surgery, hematuria, fluid intake and recurrent tract urinary tract infections can lead the provider to correctly diagnose a condition that leads to OAB as a secondary effect.

Like a good history, a focused physical exam can uncover other conditions that present with OAB symptoms. A digital rectal exam in the male patient may identity prostate pathology and prompt an evaluation of bladder outlet obstruction, especially in a patient with coincident voiding complaints. A pelvic exam in a female patient may reveal significant pelvic organ prolapse or rarely an anterior vaginal wall mass causing urinary outflow obstruction. Lower extremity edema may signify fluid retention and, especially in a patient with the primary complaint of nocturia, may serve as an explanation for the patient's symptoms.

Point of care testing has a role in evaluation of a patient presenting with OAB complaints (14). An urinalysis should be obtained to rule out infection and microscopic hematuria. A post-void residual measured ultrasonographically or with an in-and-out catheterization is helpful for ensuring that bladder emptying is adequate, and that urinary retention is not playing a role in the patient's complaints. A frequency-volume chart can be particularly helpful as it can outline fluid intake, average and maximum bladder volumes, and timing of voids. These parameters can be useful for diagnosing conditions such as polydypsia and polyuria that can masquerade as OAB. More advanced diagnostic modalities such as urodynamics, cystoscopy or upper tract imaging are only necessary when the diagnosis is uncertain or if there is a high suspicion for another condition (14).

Treatment options for OAB tend to be divided by “lines of therapy” that correspond to different levels of invasiveness ranging from least to most invasive. Lifestyle modification and pelvic floor physical therapy are the tenets of the first line of therapy and include techniques such as timed voiding, urge suppression, fluid reduction, avoidance of certain bladder irritants and pelvic floor muscle strengthening (15, 16). Second line therapy, which will be discussed in greater detail in this review, consists of drug therapy with anticholinergics and/or beta-3 agonists. Third line therapies include intravesical botulinum toxin injection, sacral neuromodulation, and percutaneous tibial nerve stimulation. While treatment should ideally be gradually escalated from least to most invasive, different therapeutic modalities can be combined to achieve the desired symptomatic control. In rare cases when the first three lines of therapy are not adequate, more invasive treatment options such as bladder augmentation or urinary diversion can be considered (17).

Both objective and patient-reported instruments can be used to assess treatment response and efficacy. Frequency-volume charts can document changes in the number of diurnal and nocturnal voids, incontinence episodes, pad changes etc. Although there is no definition of objective treatment success in OAB, most studies examining new therapies take a 50% reduction in voids or incontinence episodes to signify that the therapy is effective (18). Practically, patient-reported outcomes are more relevant to assessing treatment success. Instruments such as the Patient Global Improvement (PGI) scale and any of the validated OAB questionnaires can be used to quantify the patient's sense of improvement. The additional advantage of validated questionnaires is the ability to follow OAB symptoms using consistent instruments over time.

**Table:** 

Key Points
**Treatment principles** Treatment options for OAB are divided by “lines of therapy” based on levels of invasiveness; First line includes lifestyle modifications and pelvic floor physical therapy; Second line consists of drug therapy with anticholinergics and/or beta-3 agonists; Third line includes intravesical botulinum toxin injection, SNM* and PTNS**; Treatment should ideally escalate from least to most invasive, but different modalities can be combined if single-therapy approach is not successful.

## ANTIMUSCARINICS

### a) Mechanism of action and pharmacological properties:

Detrusor contractions are triggered mainly by acetylcholine (ACh)-induced stimulation of muscarinic receptors on bladder smooth muscle (19). ACh antagonists which bind to these receptors inhibit normal and involuntary detrusor contractions. Muscarinic receptors are also present in bladder urothelium and suburothelium, and there is a suggestion that Ach release by the urothelium and by suburothelial cholinergic fibers may influence detrusor function (20, 21).

Of the five muscarinic receptor subtypes (M1 to M5) that have been identified in humans, the M2 is the predominant subtype, but M3 receptors mediate most bladder smooth muscle contraction (19, 22).

Antimuscarinic agents (AM) differ in molecular size, charge and lipophilicity. They are categorized as tertiary or quaternary amines. Tertiary agents have higher lipophilicity and less molecular charge, both of which along with small molecular size increase the passage through the blood-brain barrier (23). They include atropine, darifenacin, fesoterodine, oxybutynin, propiverine, solifenacin, and tolterodine. Quaternary agents such as propantheline and trospium have greater molecular charge and less lipophilicity with limited passage into the central nervous system (CNS) and lower risk of CNS side effects (24).

Many antimuscarinics are metabolized by the P450 enzyme system to active and/or inactive metabolites (25). Because of the metabolic conversion there is a risk for drug interactions, that may result in reduced or increased plasma concentration of the antimuscarinic and or the interacting drug. Antimuscarinics and/or their active metabolites may be excreted in urine with the potential to affect the urothelial muscarinic receptors, but this has not been shown to improve their efficacy (26).

### b) Antimuscarinic agents:

#### Darifenacin:

Darifenacin has selectivity for M3 receptors which is the more important receptor for detrusor contraction, which might increase efficacy and reduce adverse events associated with the antagonism of other receptor subtypes (27). Darifenacin is actively removed from the brain through a protein-mediated transporter system, which was also shown for trospium and fesoterodine (23).

#### Fesoterodine:

Fesoterodine is a non-subtype selective muscarinic receptor antagonist (28). It is a pro-drug promptly metabolized to 5-hydroxymethyl tolterodine (5-HMT), the same active metabolite of tolterodine, by ubiquitous esterases (29).

#### Imidafenacin:

Imidafenacin is a muscarinic antagonist with greater affinity for the M3 and M1 receptors than the M2 receptor (30). The drug is primarily metabolized in the liver by cytochrome P450 enzyme CYP3A4 (31). Clinical studies have been performed mainly in Japan, and the drug is not available in Western countries (32).

#### Solifenacin:

Solifenacin has modest selectivity for the M3 receptor over the M2 and marginal selectivity over the M1 receptors (33). It is metabolized in the liver utilizing the cytochrome P450 enzyme system (CYP3A4), but a modest percentage undergoes renal excretion without additional metabolism raising the possibility that it could also work from the luminal side of the bladder (34, 35).

#### Oxybutynin:

Oxybutynin is the oldest agent in use for OAB and remains as either the first or second most prescribed agent in many countries (36–39). It is an antimuscarinic agent that also has strong independent musculotropic relaxant activity and local anesthetic activity (40, 41). It is metabolized primarily by the CYP system into its primary metabolite, N-desethyl-oxybutynin (DEO) (42). It has IR and ER oral formulations as well as a transdermal delivery system and a transdermal gel formulation (43–45). Transdermal administration alters the metabolism of the drug, reducing the rate of dry mouth in comparison with the oral administration. Most common adverse events are pruritus and erythema at the application site (46).

#### Propiverine:

Propiverine is a nonselective antimuscarinic agent with musculotropic smooth muscle relaxant activity (47). It also has calcium antagonistic properties and alpha(1)-adrenoceptor antagonist effects, but the importance of these for the clinical effects of this agent is not known (48).

#### Tolterodine:

Tolterodine has a major active metabolite, 5-HMT, which significantly contributes to the therapeutic effect (49). It does not have muscarinic subtype selectivity, but experimental studies indicate it has functional selectivity for the bladder over the salivary glands (50, 51). It is available in immediate release (IR) and extended release (ER) formulations. The ER formulation offers more stable blood levels which appears to improve efficacy and tolerability (52). There appears to be a very low incidence of cognitive side effects, which is due to its low lipophilicity, minimizing penetration into the CNS (24, 29).

#### Trospium:

Trospium is a hydrophilic quaternary amine with limited ability to cross the blood-brain barrier (23, 53). This results in minimal chance of promoting cognitive dysfunction (54, 55). Trospium does not have muscarinic subtype selectivity and undergoes negligible metabolism by the hepatic cytochrome P450 system, offering a lower potential for drug-drug interactions which may be an advantage especially in the context of polypharmacy (56). It is mainly eliminated unchanged in the urine by renal tubular secretion but it is unknown whether this contributes to its clinical efficacy (57).

### c) Efficacy of antimuscarinics for OAB:

Various antimuscarinic agents have been extensively evaluated for the treatment of patients with OAB and it has been shown that they are more effective than placebo in improving continent days, mean voided volume, urgency episodes, and micturition frequency (58). They also improve health-related quality of life (HRQoL) (59, 60). Consistent with this, they remain as the most widely used treatment for urgency and urgency incontinence and current guidelines from different scientific organizations strongly recommend their use for patients with OAB (14, 61, 62).

All commercially available antimuscarinic agents improve symptoms with comparable efficacy, but with different tolerability profiles (63, 64). There are not enough well-powered studies comparing the different anticholinergic drugs and no definite conclusions can be drawn regarding the superiority of one agent over the others in terms of efficacy. Although some studies and meta-analyses may show superiority of one agent over the other in specific aspects, the studies from which these results are driven were not designed to compare the agents and/or the magnitude of the differences have little clinical impact. Because each drug has specific pharmacologic properties and the dosing schedule differ, and because patients may have medical comorbidities and use other medications, it is essential to individualize the medical treatment of patients with OAB (65). Since there is scant evidence of superiority of any specific agent, we will not discuss studies comparing antimuscarinic agents. Table-1 displays the available antimuscarinic agents, their dosing schedule and efficacy assessment based on the modified Oxford Centre for Evidence Based Medicine (https://www.cebm.net/2009/06/oxford-centre-evidence-based-medicine-levels-evidence-march-2009). We only included antimuscarinic drugs that have level of evidence 1 and grade of recommendation A or B. Dose escalation of antimuscarinic drugs may be appropriate in selected patients to improve treatment effect although higher rates of adverse events can be expected.

**Table 1:** Antimuscarinic agents for adults with OAB.

	Starting dose regimen*	Dose escalation*
Darifenacin	7.5mg	15mg
Fesoterodine	4mg	8mg
Imidafenacin	0.1mg bid	0.2mg bid
Oxybutynin**	10mg ER or 5mg IR bid or tid	115-20mg ER or 5mg qid
Propiverine**	30mg ER or 15mg IR bid	45mg ER or 15mg IR tid
Solifenacin	5mg	10mg
Tolterodine	4mg ER or 2mg IR bid	Not evaluated
Trospium	60mg ER or 20mg bid	Not evaluated

* Recommended initial dose for adults with no liver or renal function impairment; Unless noted, regimens are once-daily dosing; bid: twice a day; tid: three times a day; IR: immediate release; ER: extended release;

** Agents with mixed mechanism of action but predominant antimuscarinic action.

### d) Adverse effects and contraindications:

Because muscarinic receptors are present throughout the body and there are no antimusarinic with significant selectivity for the lower urinary tract, adverse effects of treatment are common. The most common adverse events are dry mouth and constipation. In addition, blurred vision, pruritus, tachycardia, somnolence, impaired cognition, and headache may occur. In general, higher doses of any antimuscarinic are associated with higher rates of adverse events. Using a network meta-analytic approach, Kessler et al. assessed all reported adverse events of the currently used antimuscarinics (66). Their analysis included 69 studies with a total of 26.229 patients. Studies compared at least one antimuscarinic for treating OAB with placebo or with another antimuscarinic with an average treatment duration of 8 weeks. Considering the currently used starting oral dosages, a similar adverse event profile was observed for darifenacin, fesoterodine, propiverine, solifenacin, tolterodine and trospium chloride but not for oxybutynin, which demonstrated the highest adverse event rates. Immediate-release antimuscarinics have a greater risk of side effects than extended release (ER) formulations because of differing pharmacokinetics (67, 68). The concomitant use of antimuscarinics with medications with anticholinergic properties may increase the risk of side effects (69). The risk may also be increased in patients with impaired renal or liver function depending on the pharmacokinetics of the drug (65). Contraindications for the use of antimuscarinics include urinary retention (including post-void residuals >150-200mL), gastric retention, decreased gastrointestinal motility conditions, and narrow-angle glaucoma. The distinction between open-angle and narrow-angle glaucoma is essential and may warrant referral to an ophthalmologist (70).

- Urinary retention and antimuscarinics: The inhibitory effect of antimuscarinics on detrusor contraction could worsen bladder emptying and contribute to urinary retention. Contrary to this hypothesis, the network meta-analytic study by Kessler et al. showed similar urinary tract related adverse events between antimuscarinics and placebo (66). The current understanding is such that the dose range used for beneficial effects in OAB is lower than that needed to produce a significant reduction in the voiding contraction (70). Consistent with this, the use of antimuscarinics in association with an alpha-blocker in men with BPH and a moderately enlarged prostate (up to 75g) has been shown to be safe even in patients with a post-void residual of up to 150mL (71). Yet, monitoring PVR in patients with BPH and/or incomplete bladder emptying is recommended.
- Because muscarinic receptors are abundant in the CNS and play a role in cognitive functions such as memory, problem solving and vigilance, the use of antimuscarinics may be associated with neurological adverse events especially in elderly patients and those with neurological conditions (72, 73). Although most trials with antimuscarinics for the treatment of OAB did not show significant neurological side effects associated with this class of medications, it must be emphasized that cognitive impairment has not been evaluated in most studies (66). Solifenacin, trospium, and darifenacin have been shown to carry a lower risk of cognitive effect than oxybutynin, with little or no cognitive risk to otherwise healthy older adults with OAB (55, 74).

Recent studies have shown an association between the cumulative use of medications with anticholinergic activity and the risk of dementia (75–77). It is speculated whether this could be a direct effect of using anticholinergics or due to a selection bias where these drugs are used in individuals with higher potential for developing dementia. As this association continues to be investigated, there have been recommendations for avoiding the use of anticholinergics in the elderly population, including by the American Geriatrics Society in their most recent Beers Criteria document (78) and also by Fit for the Aged (FORTA) criteria, another system for prescribing appropriate medications for older persons (79).

The use of antimuscarinics in high-risk individuals other than elderly subjects should also be avoided. Finally, the clinician should consider reconciling the medications of a given patient to reduce anticholinergic burden (69, 75, 80).

### e) Adherence and persistence with antimuscarinics:

Typically, adherence in clinical trials is much higher than in real world clinical practice (68). Studies from real world experience have reported average adherence periods of few weeks to few months with different antimuscarinics. Recent studies from Canada and the United Kingdom have confirmed low persistence rates for all the antimuscarinic agents. In the study from UK, the median time to discontinuation varied from 30 to 78 days (36). In the Canadian study, median time to discontinuation for the different antimuscarinics varied from 75 to 108 days, with around 20% of patients persisting on medication for 12 months (37).

### f) Transdermal formulations:

Transdermal formulations of oxybutynin have the advantage of bypassing the hepatic metabolism by CYP3A4 enzymes, hence increasing the bioavailability of oxybutynin and lowering the serum concentration of DEO, the metabolite that is mainly responsible for side effects associated with this agent (81, 82). It may result in greater tolerability for the patient while maintaining efficacy (82–84). The risk of dry mouth is reduced to approximately 7%, significantly lower than observed for oral formulations (85).

Transdermal oxybutynin formulations have a long half-life which make them appropriate for patients who have poor adherence to oral treatment (82). As these formulations bypass metabolism by CYP3A4 enzymes in the liver, they may be a better option for patients at risk for potential drug-drug interactions (81, 82, 86).

Transdermal formulations are administered according to their delivery system which may be a gel or a patch with different dosing regimens. They must be placed on dry, intact skin, and patients should be informed to avoid strenuous activity or bathing immediately after placement (81, 82, 86). Transdermal gel can be applied directly to the skin and should be covered with clothing to avoid transmission to close contacts. Transdermal application may cause skin reactions at the application site like erythema, rash, and pruritus. Although these reactions are usually minor, they occur in between 3% and 32% of the patients and may lead to treatment discontinuation (82). The safety of transdermal formulations has not been well established in pediatric patients.

It should be noted that at the time of this publication, access to transdermal oxybutynin has been limited, and certain pharmacies may not carry the medication. Furthermore, the cost to the patient is another possible limiting factor.

### g) Intravesical antimuscarinics:

Intravesical administration of oxybutynin has been used by patients with neurogenic lower urinary tract dysfunction who perform intermittent catheterization (87). Dosage for children with neurogenic voiding dysfunction varies according to patient's weight and no specific formulation has been approved. Different oxybutynin concentrations have been used, which are either prepared from oral formulations (liquid or crushed tablet in solution) or manufactured in a compounding pharmacy. Several non-controlled studies have demonstrated the efficacy of this therapy in a variety of patients with neurogenic bladder (88–90).

**Table:** 

Key Points
**Antimuscarinics (AM)** AM act mainly by blocking M3 receptors; Because there are no AM with significant selectivity for the bladder, adverse effects (AEs) of treatment are common; AM differ in molecular size, charge and lipophilicity; Quaternary AM have greater molecular charge and less lipophilicity which limit their passage into the central nervous system; Many AM are metabolized by the P450 enzyme system which may affect the plasma concentration of the AM and that of an interacting drug; All commercially available AM improve OAB symptoms and quality of life with comparable efficacy, but different tolerability profiles; The most frequent AEs are gastrointestinal, with dry mouth as the most common; Considering the starting oral dosages, a similar AE profile was observed for most AM, with the exception of oxybutynin which demonstrated higher AE rates; Immediate-release AM have a greater risk of side effects than extended-release formulations; Recommended AM dosages do not significantly inhibit voiding contraction; AM should be avoided in the elderly population since the cumulative use of medications with anticholinergic activity may be associated with the risk of dementia; Persistence in treatment with AM is low, with only 20% persisting after 1 year; Due to specific pharmacologic properties and dosing schedule, AM treatment must be individualized; Intravesical administration of oxybutynin is an option for patients with neurogenic dysfunction who perform intermittent catheterization.

## ß3-AR AGONISTS

By the end of the previous century two different groups used RT-PCR to identify a third type of Beta-adrenoceptor (β-AR) mRNA in isolated human detrusor. Now known as the β3-AR, pharmacological assays have shown that it participates in beta adrenergic-mediated bladder relaxation. The generally accepted mechanism of action of β3-AR agonists implicates the activation of adenylyl cyclase, with formation of cAMP, leading to detrusor relaxation (91). A recent study also demonstrated the expression of β3-AR in cholinergic nerve endings of the human bladder suggesting a possible role of this receptor in the modulation of acetylcholine release (92). The role of β3-AR expressed in sensory fibers and in urothelial cells still remains unclear. Outside of the bladder, β3-AR are mostly expressed in the adipose tissue, gastrointestinal tract and gallbladder, uterus and central nervous system (91).

Mirabegron became the first β3-AR agonist available for clinical practice, following FDA and EMA approval in 2012. Since then, most countries throughout the World approved it for OAB treatment. More recently a second β3-AR agonist, vibegron, was licensed for the treatment of OAB by the Japanese Heath authorities in 2018 and by the FDA in 2020 (93, 94).

### Mirabegron

Current guidelines of all scientific organizations strongly recommend mirabegron for the treatment of idiopathic OAB/LUTS. In a pooled efficacy analysis of pivotal randomized, double-blind, placebo-controlled, phase III studies mirabegron 50mg was more effective than placebo in reducing the mean number of incontinence episodes/24h, mean number of urgency episodes/24h and mean number of micturitions/24h. In addition, the percentage of dry patients was significantly higher after mirabegron 50mg (44.1%) compared with placebo (37.8%) (95).

Although the most frequent marketed dose of mirabegron is 50mg, some countries offer the β3 agonist in both 25mg and 50mg doses. Both are effective, although mirabegron 50mg shows some superiority over the lower dose. In fact, although both doses at 12 weeks were more effective than placebo for frequency and urgency incontinence control, at 4 and 8 weeks only mirabegron 50mg reached statistical superiority over placebo, suggesting a faster therapeutic effect for the higher dose (96). In addition, mirabegron was tested in elderly OAB patients. The 12-week Pillar study used a mirabegron flexible dosing regimen, starting with 25mg/day with option to escalation to 50mg/day at week 4 or 8. It showed that mirabegron is effective in patients above 65 year of age. About 50% required escalation to 50 mg, suggesting a reduced overall effect of the lower dose regimen (97).

Mirabegron and anticholinergic drugs were never compared in well-powered studies. However, in a phase III trial, tolterodine 4mg ER, used as comparator for mirabegron 50mg, provided numerically inferior reductions of urinary frequency and of incontinence episodes (98). In a large systematic review involving more than 30.000 subjects, efficacy of mirabegron 50mg in reducing frequency and urgency incontinence did not differ significantly from most anticholinergic drugs in low dose. Only solifenacin 10mg and fesoterodine 8mg provided a slightly superior effect for frequency and urgency incontinence, respectively (99). Mirabegron 50mg may be effective in OAB patients refractory to anticholinergics (100).

Mirabegron may improve the persistence of OAB patients on pharmacological treatment. UK and Canadian databases indicate that mirabegron exceeds the typical low persistence associated with anticholinergic drugs, reaching figures of 31.7% to 38% after 12 months, as opposed to 8.3 to 25.0% for the different antimuscarinics (36, 37). In the 12-month observational Believe study, involving 862 patients, 53.8% of the participants were still taking mirabegron at 12 months (101).

Mirabegron 50mg does not compromise the voiding detrusor contraction in OAB male patients. This relevant point was first shown in a small cohort of OAB male patients with urodynamically proven bladder outlet obstruction (102). In a recent placebo-controlled study involving more than 400 OAB male patients, mirabegron did not cause relevant changes in maximum urinary flow and post-void residual urine while producing a robust improvement in storage (OAB) symptoms (103).

All phase III trials showed that mirabegron has a high safety profile. Hypertension was particularly investigated despite being a selective β3-AR, in order to rule out potential activation of other β-ARs. Hypertension had similar incidence in the mirabegron and placebo arms. The incidence was high in both groups most probably due to the exacting definition of hypertension required by the regulatory authorities. The analysis of a large database involving more than 10.000 patients that participated in OAB clinical trials gives an additional strong validation of the safety of mirabegron (104). Total adverse events in mirabegron participants amount to 17.0% while in those exposed to anticholinergics was 21.4%. Rates of dry mouth and constipation in the elderly (≥75y) were the most striking differences. Appearance or aggravation of hypertension was similar across subjects exposed to mirabegron or anticholinergic drugs, except for patients ≥75y, who showed a small increase of this event (1%) compared to placebo arms. Despite these data, mirabegron remains contraindicated in patients with severe uncontrolled hypertension and a regular vigilance of blood pressure is recommended after its prescription (104). Patients exposed to mirabegron did not show any evidence of cognitive deterioration: the 12-week Pillar study which exposed patients ≥65y to mirabegron, did not find any cognitive deterioration based on the Montreal Cognitive Assessment score (105). A large population-based Canadian study which included >20.000 new users of mirabegron and >40.000 new users of anticholinergic medications (oxybutynin, tolterodine, solifenacin, darifenacin, fesoterodine, trospium) concluded that the risk of dementia was lower among those using the β3-AR agonist (77). Thus, mirabegron may be an excellent choice for elderly patients who have or are at risk of developing cognitive dysfunction. Anticholinergic drugs in these patients should be used with caution as discussed above (79).

### Vibegron

A second β3-adrenergic receptor agonist, vibegron, was recently introduced in the Japanese and North American markets for OAB treatment, following successful Phase III trials.

A 12-week Phase III trial conducted in Japan enrolled over 1000 participants, consisting predominantly of OAB wet patients (106). Subjects received vibegron 50 or 100mg, placebo, or the antimuscarinic imidafenacin, 0.1mg TID. The primary endpoint, a reduction in the number of micturitions per 24h, was met for both vibegron doses and more than 50% of the incontinent patients became dry. Interestingly, more than 40% of the subjects exposed to vibegron, in both doses, exhibited resolution of nocturia. Overall adverse events, including hypertension were similar in vibegron and placebo arms and inferior to the antimuscarinic group. Vibegron 50mg/day is approved in Japan (93).

The EMPOWUR study, also a 12-week Phase III, double-blind, placebo, and active-controlled study, enrolled a total of 1518 OAB patients (107). About three fourths of the participants had OAB wet. Subjects were randomized to vibegron 75mg, placebo, or tolterodine ER 4mg. Vibegron resulted in a statistically significant reduction in urgency urinary incontinence episodes in patients with ≥1 episodes/day and in voids/day over placebo. Vibegron-associated adverse events were mild and less frequent than in the tolterodine arm. Vibegron 75mg/day was thus approved in the U.S (94).

Both studies had an antimuscarinic drug as comparator, which demonstrated numerically inferior improvements in frequency and incontinence than those seen in the Vibegron arms. Vibegron was well tolerated. Adverse events reported in the two studies were mild and hypertension in the EMPOWUR study had an incidence of 1.7% in both the active and placebo arms.

Vibegron, in contrast to mirabegron, does not inhibit CYP2D6, a cytochrome P450 enzyme (108). How much this characteristic can contribute to decrease drug interaction between vibegron and other drugs in real life is still unclear. EAU and AUA guidelines do not mention yet recommendations for Vibegron (14, 68). However, when updated, it is expected that they will not differ substantially from those stated for Mirabegron.

**Table:** 

Key Points
**Beta-3 agonists** β3-AR agonists promote detrusor relaxation through activation of adenylyl cyclase and formation of cAMP; Mirabegron improve OAB symptoms and quality of life and is recommended by current guidelines for the treatment of OAB; Some countries offer mirabegron in both 25mg and 50mg doses; 50mg shows some superiority over lower doses; The efficacy of Mirabegron and AM were never compared in well-powered studies; Persistence in treatment with mirabegron exceeds that of AM; Mirabegron does not inhibit voiding contraction; Mirabegron has a high safety profile including for cardiovascular events; Appearance or aggravation of hypertension is similar in subjects exposed to mirabegron or AM; Yet, mirabegron is contra-indicated in patients with uncontrolled hypertension and regular vigilance of blood pressure is recommended after its prescription; Rates of dry mouth and constipation are reduced compared to AM, especially in the elderly (≥75y); Patients exposed to mirabegron did not show evidence of cognitive deterioration; Vibegron (75mg/day) is a new β3-AR agonist that has recently been approved for use in OAB patients; Hypertension is similar in subjects exposed to Vibegron or placebo; The efficacy and safety profiles of vibegron have not been compared to mirabegron and there is a scarcity of studies evaluating its use in combination with other drugs.

### Β3-AR agonists in combination with other drugs

Mirabegron and anticholinergic drugs act through distinct intracellular pathways. Thus, combination is expected to provide superior efficacy. Studies have investigated combination in an add-on practice.

In the scenario of an antimuscarinic being the first drug prescribed, mirabegron 50mg may increase efficacy while avoiding the expected adverse effects of anticholinergic dose escalation (109). OAB-wet patients not satisfied with solifenacin 5mg received mirabegron 50mg. Combination was more effective than solifenacin 10mg and caused fewer adverse events (110). In long term administration (52 weeks), the combination remained effective and safe (111).

When mirabegron is the first drug to be introduced and patients remain unsatisfied, the combination of an antimuscarinic agent at the lowest dose possible (solifenacin, propiverine, imidafenacin or tolterodine) is also an effective option. In a 52-week study the therapeutic effect of combination with each anticholinergics was effective, durable and safe (112).

The combination of mirabegron with tadalafil was also recently evaluated. The CONTACT study compared the efficacy and safety of tadalafil monotherapy 5mg/day versus the combination of tadalafil plus mirabegron (5mg/50mg/day), in 176 men with LUTS refractory to monotherapy (113). OAB symptoms were significantly improved in the combination arm without producing alarming adverse events in comparison to monotherapy.

One small single arm study evaluated the efficacy and safety of vibegron (50mg/day) add-on therapy in 42 men with persistent storage LUTS receiving either an alpha-1 blocker (22 patients) or a PDE5 inhibitor (20 patients) (114). After 12 weeks of treatment a significant improvement of storage symptoms was observed based on the decrease in the total Overactive Bladder Symptom Score. Maximum flow rate and residual urine volume did not change, and no patient discontinued vibegron because of adverse events.

**Table:** 

Key Points
**Drug combinations** Adding Mirabegron to patients unsatisfied with monotherapy with an AM provides superior efficacy; Adding an AM to patients unsatisfied with monotherapy with mirabegron is also effective; Adding Mirabegron to men with LUTS unsatisfied with monotherapy with tadalafil provides superior improvement of OAB symptoms without significant AE; The efficacy and safety of combining vibegron with other agents has yet to be shown.

## NEW DIRECTIONS

Anticholinergics and beta-3 agonists are the only two classes of oral therapeutics approved for use in OAB. However, bladder sensation, contractility and relaxation are mediated by many other receptors and neurochemical mechanisms. Some of these are being explored as potential targets for OAB. Transient receptor potential (TRP) channels are abundant in the bladder. Their activity is quite variable as they have been implicated in mechanotransduction, pain and temperature sensation (115). Because normal bladder sensation is thought to be impaired in OAB, altering afferent neural signaling via TRP receptor modulation can hypothetically change OAB symptomatology. Perhaps the best known of the TRP receptors is the TRPV1, which is desensitized by such agonists as capsaicin and resiniferotoxin. Both have shown promise in improving symptoms of neurogenic detrusor overactivity but have been rendered somewhat obsolete by the availability of intradetrusor botulinum toxin. TRPV1 agonists are not suitable in idiopathic OAB because of pain associated with their administration. On the other hand, TRPV1 inhibitors may prove to be a much more suitable option. Several TRPV1 inhibitors have been investigated in both preclinical and clinical studies (116). Although TRPV1 inhibition has not been assessed for its effect on bladder function in humans, several animal studies have demonstrated a reduction in detrusor contractility and increase in bladder capacity with oral, intravesical and intravenous TRPV1 administration. One barrier to TRPV1 inhibitor use in humans is the development of hyperthermia but newer inhibitors tested in human subjects do not seem to elicit this adverse effect (117). While TRPV1 is perhaps the best studied member of the TRP family with respect to lower urinary tract function, many other TRP receptors have been identified in the bladder including TRPV4, TRPM8, TRPA1 and TRPM4. All of these have been assessed in vitro or in animal models with variable success and investigations into their potential efficacy in OAB continue (116).

P2X3 receptors bind urothelial ATP and play a critical role in the activation of sub-urothelial sensory fibers in order to generate bladder sensation and initiate the micturition reflex. P2X3 antagonists may therefore provide a new treatment for OAB. Pre-clinical data with P2X3 receptor antagonists and P2X3 knockout-mice have shown a reduction in voiding frequency and increase in bladder volume thresholds without changing the amplitude of detrusor contractions (118). Clinical evidence from preliminary human studies showed a significant reduction in urinary urgency (119). Further clinical trials are ongoing in Europe.

The cannabinoid receptor is another potential target for OAB therapy. These receptors are present in the human bladder and urethra and, compared to healthy controls, they have been reported to be overexpressed in the detrusor and sub-urothelial layers of painful bladder syndrome and OAB subjects (120). Although the role of cannabinoid receptors in the urothelium is not fully understood, activation of these receptors is thought to decrease afferent neural signaling by decreasing the release of activating neuropeptides such as calcitonin gene related peptide (CGRP) and adenosine triphosphate (ATP) (121–123). Activation of cannabinoid receptors was found to increase bladder capacity and decrease maximal voiding pressures in an animal model study (124). Translation to human subjects has been primarily explored in multiple sclerosis patients. In a 2016 study of 15 patients, cannabidiol/tetrahydrocannabinol (THC/CBD) oral-mucosal spray administered for four weeks was found to improve overactive bladder symptoms. Although not statistically significant, there was a modest increase in maximum bladder capacity and bladder volume at first desire to urinate (125). Obvious safety concerns exist for using cannabinoid receptor agonists in able-bodied OAB subjects but development of selective activators that do not have systemic effects is a promising avenue for the future.

Potassium channels are widely distributed throughout the bladder and play an important role in maintaining detrusor muscle depolarization and repolarization. A recent Phase I study of injectable potassium channel gene plasmid vector demonstrated good safety and modest improvement in urgency and voiding episodes in able-bodied OAB subjects (126). Despite these promising results with an injectable formulation, it is unlikely that sufficiently selective oral potassium channel agonists will be developed in the near future. There is a myriad of other potential molecular targets for OAB therapy. These include purinergic receptor blockers, TGF-beta pathway modulators, and Rho-kinase inhibitors, among others. These targets are in the nascent stage of development and only preclinical or in vitro studies have investigated their usefulness in correcting bladder dysfunction (127).

**Table:** 

Key Points
**New directions** Lower urinary tract sensation and contractility are mediated by a multitude of mechanisms and receptors. Some of these are being investigated as potential targets for novel oral therapies for OAB; Altering afferent bladder signaling may be a novel approach to OAB therapy; Agonists and inhibitors of pain and mechanotransduction receptors such as TRPV and cannabinoid receptors are currently in preclinical and clinical studies and have shown some promise in certain patient populations.
